# ﻿Biodiversity and taxonomy of fishes in Taiwan and adjacent waters

**DOI:** 10.3897/zookeys.1220.140735

**Published:** 2024-12-09

**Authors:** Hsaun-Ching Ho

**Affiliations:** 1 Department and Graduate Institution of Aquaculture, National Kaohsiung University of Science and Technology, Kaohsiung, Taiwan National Kaohsiung University of Science and Technology Kaohsiung Taiwan; 2 Australian Museum, Sydney, Australia Australian Museum Sydney Australia

## ﻿

Taiwan is situated in the northwestern Pacific Ocean and is surrounded by the South China Sea, the Okinawa Trough, the Philippine Sea and the Taiwan Strait. The region has a remarkably high fish diversity, comprising more than 3,400 species, which is approximately 1/10 of the world’s total fish species. Over the past two decades, significant research efforts have been dedicated to the study of fish diversity and taxonomy in Taiwan, with a particular focus on Chondrichthyes, eels (Anguilliformes), and various deep-sea fish species. As a result, more than 200 new fish species have been described in Taiwan, and a few hundred new records have been added to the Taiwanese fish fauna. These findings have significantly enhanced our understanding of the fish species in Taiwan.

In this special issue, we invited all researchers who have been studying the fishes from Taiwan and adjacent areas to contribute their new findings. A total of seven species are described as new to science, including a new jawfish (Su and Ho 2024), a new beardfish (Fan et al. 2024), four snake eels (Hibino and Ho 2024; Hibino et al. 2024a, 2024b); and a new moray from Indo-west Pacific (Huang et al. 2024); all of which were collected from Taiwan. Lin and Han (2024) studied the species diversity of freshwater glass eel and increased the number of *Anguilla* species to seven. Ho and Kawai (2024) verified two barracudina species and settled two nominal species. Su et al. (2024) re-described a rare deep-sea beryciform fish from Taiwan. Koeda and Bessho-Uehara (2024) documented ten species in the genus *Pempheris* from Taiwan and Japan, five of which were found in Taiwan. Hibino and Ho (2024) documented two new recorded *Yirrkala* species from Taiwan. Finally, Ng et al. (2024) provide a remarkable checklist of fishes collected from Dongsha Island, which includes a list of 13 recently added species and 89 species recorded from Dongsha Island for the first time, with many of them being new to Taiwan; in addition, there are 35 species without a name, and many of them are likely undescribed.

With the publication of 11 valuable papers, we further improve the fish fauna of Taiwan. It is notable that the fish diversity in Taiwan is still rapidly increasing, and we may expect that more fishes will be found in the near future.

Special thank to all authors for their contribution and three guest editors for taking care of the manuscripts.

## ﻿Additional information

### Author ORCIDs

Hsaun-Ching Ho https://orcid.org/0000-0003-1154-601X

### Data availability

All of the data that support the findings of this study are available in the main text.
